# The Efficiency of Fluoride Bioactive Glasses in Protecting Enamel Surrounding Orthodontic Bracket

**DOI:** 10.1155/2021/5544196

**Published:** 2021-03-24

**Authors:** Mona Aly Abbassy, Ahmed Samir Bakry, Robert Hill

**Affiliations:** ^1^Department of Orthodontics, Faculty of Dentistry, King Abdulaziz University, Jeddah, Saudi Arabia; ^2^Alexandria University, Alexandria, Egypt; ^3^Operative and Esthetic Dentistry Department, Faculty of Dentistry, King Abdulaziz University, Jeddah, Saudi Arabia; ^4^Conservative Dentistry, Alexandria University, Alexandria, Egypt; ^5^Physical Sciences in Relation to Dentistry, Institute of Dentistry, Dental Physical Sciences Unit, Queen Mary University of London, London, UK

## Abstract

**Objectives:**

The aim of this study was to evaluate the protective effect of using four different fluoride bioactive enamel sealers against an acidic erosion challenge.

**Materials and Methods:**

A sample of 50 freshly extracted sound upper premolars had their buccal surface bonded to 50 orthodontic brackets using Transbond PLUS color change adhesive; the first four groups had four compositions of fluoride bioactive glasses based on 37 mol% SiO_2_, 43.9-53.9 mol% CaO, 6.1 mol% P_2_O_5_ and CaF_2_, and 0-10 mol% of Na_2_O applied to their surfaces and the fifth group served as control (which was not treated by any bioactive sealer). All specimens were challenged by 1% citric acid for 18 minutes which was stirred by a magnetic stirrer. The enamel surfaces next to the orthodontic brackets were examined by SEM. The Wilcoxon signed-rank test was used to compare the area covered by the fluoride bioactive pastes before/after erosion (*p* < 0.05). Samples from the layer formed on top of the examined teeth were tested before/after erosion to be examined by the attenuated total reflectance Fourier-transform infrared spectroscopy (FTIR/ATR).

**Results:**

The FTIR/ATR test showed that fluoride bioactive pastes' applications resulted in the formation of a hydroxyapatite-rich layer; the SEM analysis showed that the aforementioned layer significantly resisted erosion challenge when compared to the control group (*p* < 0.05).

**Conclusions:**

Fluoride bioactive pastes can efficiently protect the enamel surfaces next to orthodontic brackets from acidic erosion challenges.

## 1. Introduction

Increase of caries prevalence among orthodontic patients is one of the major challenges in the dental field [1, 2] which may be attributed to the rapid formation of cariogenic biofilms around the irregularities of the orthodontic bands, brackets, and other attachments of fixed orthodontic appliances [1, 3]. Adding to the complexity of the aforementioned problem is the high rate of consuming low-pH soft drinks observed in populations inhabiting hot regions of the world [4] which may convert the early enamel lesions [5] into erosive lesions that can be only treated by restorative procedures [4].

Minimal intervention concept which is currently accepted in the dentistry field dictates that early caries lesions should be remineralized to avoid their progression and alleviate the need for removing caries lesion by the surgical intervention [6, 7]; thus, it may be suggested that there is a necessity to develop new category of materials capable of effectively remineralizing and protecting early caries lesions.

In orthodontic field, literature showed controversial results regarding the strategies employed for protecting enamel around orthodontic brackets against various acidic attacks. On the one hand, an *in vitro* research showed excellent results when bonding orthodontic brackets with fluoride-releasing bonding agents [8]; on the other hand, a comprehensive clinical study using the fluoride-releasing cementing agents showed no advantages [9].

In operative field, many methods were suggested for enamel protection which included the use of nanoparticles to modify the cariogenic oral flora in orthodontic patients, [10] the use of casein phosphopeptides-amorphous calcium phosphate [7], and the use of antiseptics as chlorhexidine [5], probiotics, polyols, and resin infiltration [10, 11].

Another strategy for preventing the development of acidic erosive lesions involved the use of orthodontic sealants which are resin materials that merely act as a mechanical barrier to protect the prone areas of enamel adjacent to the orthodontic bracket from acidic attacks [12]. However, these agents have a minimal remineralizing effect [13]. The application of bioactive materials as an alternative to the aforementioned resinous-based sealers showed promising results [14, 15] due to their ability to remineralize enamel [15–19]. Moreover, these materials showed good biocompatibility to pulp cells [20] and were resistant to the brushing abrasion [21].

In the current study, novel pastes based on different variations of fluoride bioactive glasses were tested for their ability to protect enamel against erosive challenge. The null hypothesis adopted in the current study was that the fluoride bioactive glass pastes would not exert any protective effect on the enamel surface against an erosive challenge.

## 2. Materials and Methods

50 extracted sound premolar teeth were collected from the oral surgery department after obtaining the permission of the ethical committee of the faculty. The teeth were hand scaled from any calculus or soft tissues. The teeth were stored in 0.1% thymol till the start of the experiment according to the guidelines approved by University and in accordance with the principles of the Declaration of Helsinki and its later amendments or comparable ethical standards. The number of specimens assigned to each group was adopted according to the threshold for significance which was set at 0.05 and means and standard deviation obtained in a pilot study and a previously conducted research [14], and the power of test which was set at 80%. Randomization of the specimens was done using a computer program (Excel 2007, Microsoft, Redmond, WA, USA). All teeth were examined by a light microscope to exclude any teeth having cracks, restorations, demineralization, or any defects. Intra- and interexaminer calibrations were conducted before actual recording of the obtained results.

### 2.1. Glass Synthesis and Characterization

Bioactive glasses (SiO_2_–P2O_5_–CaO–CaF_2_–Na_2_O) having a varying content of of Na_2_O (ranging between 0 and 10 mol% in exchange for CaO.) were melted in an electric oven according to temperatures listed in Table 1. The components of the glass were purchased from the following manufacturers; SiO_2_ (Prince Minerals Ltd, Stoke-on-Trent, UK); P_2_O_5_, CaCO_3_, Na_2_CO_3_ and CaF_2_ (Sigma-Aldrich, Gillingham, UK). The experimental glasses were melted for 90 minutes then quenched into water to prevent crystallization of the glass. After drying, the glasses were pulverized and sieved to obtain glass particle sizes ranging between 38 and 80 *μ*m.

The differential scanning calorimetry (DSC, Stanton Redcroft DSC1500, Rheometric Scientific, Epsom, UK) was utilized in determining the glass transition (*T*_g_, defined as the onset of the transition area) and crystallization temperature (*T*_x_, defined as crystallization peak temperature) of the glasses.

### 2.2. Bracket Bonding

All specimens were covered with a nail varnish except the buccal surface which was left uncovered. Etching of enamel bonding sites with 37% phosphoric acid was performed for 15 seconds for all teeth, and then rinsed with air-water stream, followed by thorough drying. Transbond™ XT Primer (3M Unitek, USA) was applied to etched surfaces then gently dried for 5 seconds. A small amount of Transbond PLUS color change adhesive (3M Unitek, USA) was dispensed onto the base of orthodontic brackets (Unitek™ Gemini Metal Brackets, 3M Unitek, USA) which were placed on tooth surfaces and adjusted to final position. Brackets were then bonded to the designated area, and excess adhesive was removed carefully using scalpel under microscopic observation, and then, light was cured for 10 seconds mesially and 10 seconds distally.

### 2.3. Clinical Simulation Model

**Figure 1:**
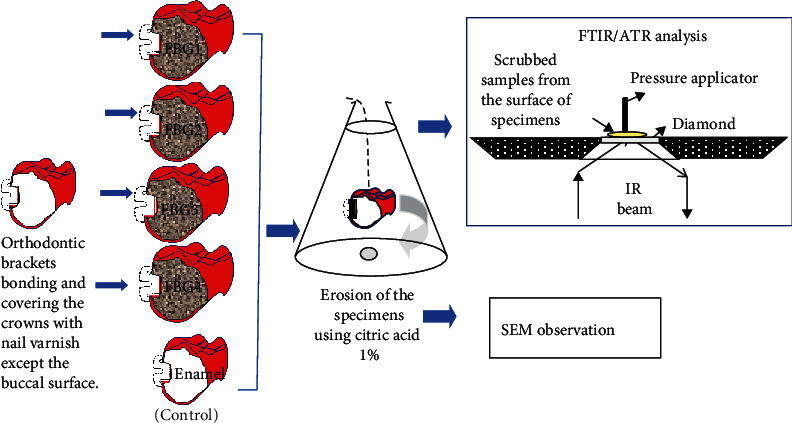
Schematic illustration for the experimental procedures.

**Figure 2:**
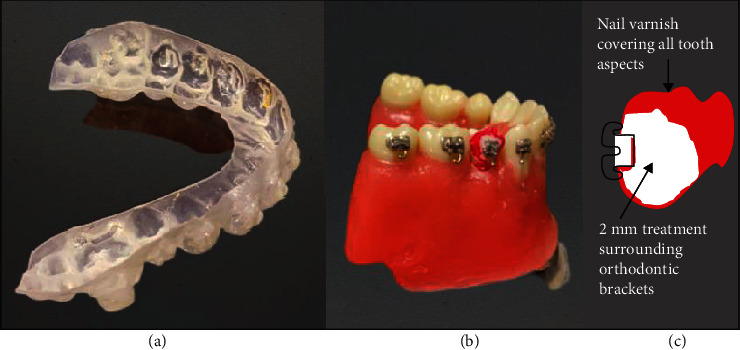
Clinical simulation model. (a) The premolar tooth was mounted on typodont, and brackets were bonded. (b) Nail varnish applied on all aspects of experimental teeth leaving 2 mm of treatment window. (c) Vacuum trays constructed on the model for applying the tested material.

The summary of the experimental procedures is illustrated in Figure 1. The bonded premolar teeth were fixed onto a dental model (Nissin Dental, Tokyo, Japan) having artificial resin teeth bearing orthodontic brackets to complete the dental arch (Figure 2). The examined teeth had nail varnish applied onto all aspects of tooth leaving a treatment window of 2 mm surrounding the orthodontic brackets. Areas of the brackets on the casts were blocked with blockout wax to allow for space needed for the application of the tested materials. A polypropylene sheet (Easy-Vac Gasket, 3A MEDES, Korea) was vacuum-adapted to each cast with a vacuum-forming machine (Henry Schein, Henry Schein Inc., NY, USA) (Figure 2). From the vacuum adapted sheet, individual trays were made to fit onto the tooth surfaces to cover the complete arch of the teeth on the model and were trimmed to be approximately 1 mm above the gingival margin.

### 2.4. Tested Material Application

The selected teeth were divided into five equal groups (*n* = 10). The specimens were treated with 4 compositions of fluoride-containing bioactive glasses (FBG1-4), and the last group (control group) received no treatment (orthodontic brackets were bonded to teeth of this group but no material was applied). One of the researchers conducted the randomization and application of the material, while the other researcher did not participate in the randomization process and conducted the SEM and the FTIR examinations. Resin materials used in this study are summarized in Table 2.

**Figure 3:**
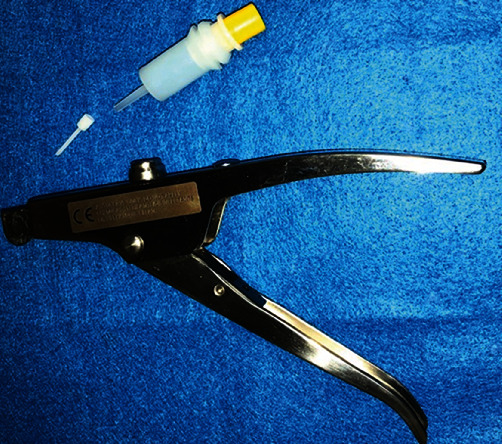
Capsule containing two chambers for powder and liquid components of the bioactive sealer. The applicator was used to express the paste outside the mixing capsule.

Fluoride bioactive glass compositions (FBG1-4) are presented in Table 1. 0.1 g of each type of the FBGs was dispensed in a chamber in a special capsule, while 0.2 ml of phosphoric acid was added to another chamber in the aforementioned capsule which is separated from the powder chamber by a thin adhesive film (Figure 3). Upon application of the FBG pastes, a cylindrical piston was pressed to rupture the tiny film and make the powder in contact with the phosphoric acid. The capsule was placed in an amalgamator (Rock-mix, Dentmark, China) to mix the content of the capsule. An applicator was used to extrude the paste outside the capsule and apply it around the orthodontic brackets as described previously [14–22]. The pH of the tested pastes was pH 2 ± 0.2 which increased steadily to reach pH 4 after 30 minutes [16]. The customized trays had small parts of the mixed pastes applied into its reservoirs and then placed onto the bonded brackets [7, 14–22]. The dental models were submerged in distilled water for one hour and kept in an incubator at 37°C. After that, the excess bioactive glass material was removed with a stream of air-water spray as was described previously [15, 18, 22].

### 2.5. Erosive Challenge

The bonded premolars were dislodged from the dental models and were examined by SEM, and the other half was suspended by a wire as demonstrated in Figure 2 in a beaker having 250 ml of 1% citric acid for 18 minutes [23] that was continuously stirred by a magnetic stirrer at room temperature to simulate the erosion challenge of ingesting the erosive soft drinks by patients [14, 22].

### 2.6. Scanning Electron Microscope Top Surface Examination

Specimens before/after the erosive challenge from each group were examined by the SEM (JCM-6000 NeoScope, JEOL, Akishima, Japan). All specimens were gradually dehydrated in an ascending ethanol series (50–100%). The specimens were fixed by adhesive carbon tape to the metallic stage of the SEM. The specimens were examined under low vacuum at 1000x magnification. The layer formed on top of the examined enamel surfaces after application of the various types of the fluoride bioactive glasses together with the control group was examined under the SEM.

### 2.7. FTIR/ATR Analysis

The infrared spectra of the layers formed on the treated enamel for all groups were examined before/after the acidic challenge. The layers were examined by gently scraping it using a sharp scalpel according to the method described previously [24]. The FTIR spectra were obtained by a FTIR spectrometer (Nicolet iS5 FTIR, Thermo Electron Scientific Instruments LLC, Madison, WI, USA) equipped with an ATR attachment. The obtained samples from the treated surfaces were pressed onto the face of a diamond of the ATR attachment [24]. The spectra were obtained under the following conditions: multiple reflections, 500–4000 cm^−1^ range, 4 cm^−1^ resolution, and entrance angle of 45° (Figure 2).

### 2.8. Statistical Analysis

The areas of the enamel surfaces that were demarcated by nail varnish were calculated for each specimen at the beginning of the experimental procedures. After concluding the application of the tested bioactive materials and the erosion challenge experiment, SEM images were obtained. The calculation of the tested areas on the SEM images was compared to the actual dimensions of the area measured, and any errors were corrected. When the bioactive material was covering all parts of the tested (demarcated area), so the degree of coverage was considered 100%. The percentages of coverage by the bioactive materials for the enamel surfaces before and after the erosion challenge were compared using the Wilcoxon signed-rank test [16]. Differences were considered statistically significant at the level of 0.05. Software utilized was SPSS (v24, IBM, Armonk, USA).

## 3. Results

### 3.1. Scanning Electron Microscope Top Surface

**Figure 4:**
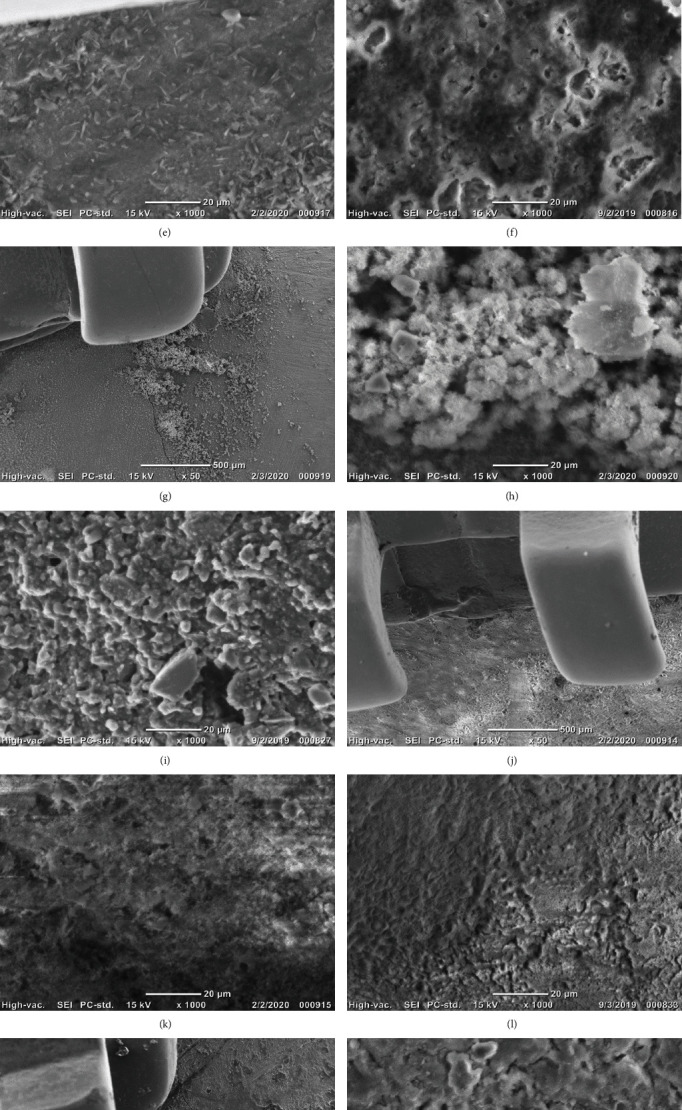
SEM observation for the top view of the specimens. Low magnification (a) and high magnification (b) top surface view for the control group (before erosion challenge) showing smooth enamel surface next to the orthodontic bracket. (c) High magnification for the control group (after erosion challenge) showing rough enamel surface with obvious boundaries of the enamel prisms. Low (d) and high (e) magnification for the FBG1 group (before erosion challenge) showing crystalline structures covering the areas next to the orthodontic bracket. (f) High magnification for the FBG1 group (after erosion challenge) showing resistance of the formed layer to erosion challenge. Low (g) and high (h) magnification for the FBG2 group (before erosion challenge) showing crystalline structures covering the areas next to the orthodontic bracket. (i) High magnification for the FBG2 group (after erosion challenge) showing resistance of the formed layer to erosion challenge. Low (j) and high (k) magnification for the FBG3 group (before erosion challenge) showing crystalline structures covering the areas next to the orthodontic bracket. (l) High magnification for the FBG3 group (after erosion challenge) showing resistance of the formed layer to erosion challenge. Low (m) and high (n) magnification for the FBG4 group (before erosion challenge) showing crystalline structures covering the areas next to the orthodontic bracket. (o) High magnification for the FBG4 group (after erosion challenge) showing resistance of the formed layer to erosion challenge.

**Figure 5:**
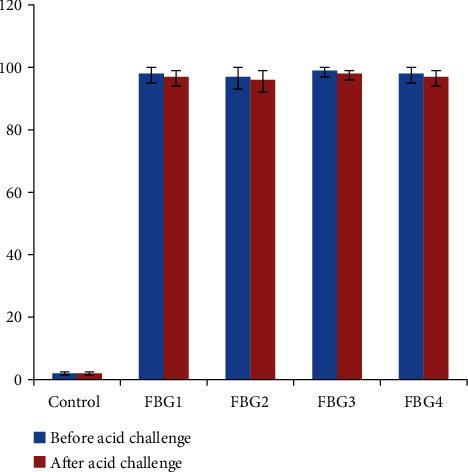
Degree of coverage for the enamel surface by the interaction layer. Control specimens showed significant difference with the rest of the specimens, while the FBG specimens did not show any statistically significant differences (*p* < 0.05).

The enamel surface that was not treated by any bioactive glass (control) before exposure to the erosion challenge revealed a smooth surface (Figures 4(a) and 4(b)). The (control) group (Figure 4(c)) after exposure to the erosion challenge revealed rough enamel surface in which the borders of the enamel prisms were evident due to the erosion challenge. Fluoride bioactive-treated specimens showed the coverage of the whole surface by crystalline structures (Figures 4(d)–4(o)) before/after exposure to the acid challenge. The Wilcoxon signed-rank test showed that the formed interaction layer on the enamel surface was not significantly changed after the erosion challenge (*p* < 0.05) for the FBG1-FBG4 groups (Figure 5).

### 3.2. Attenuated Total Reflectance Fourier-Transform Infrared Spectroscopy (FTIR/ATR) Analysis

**Figure 6:**
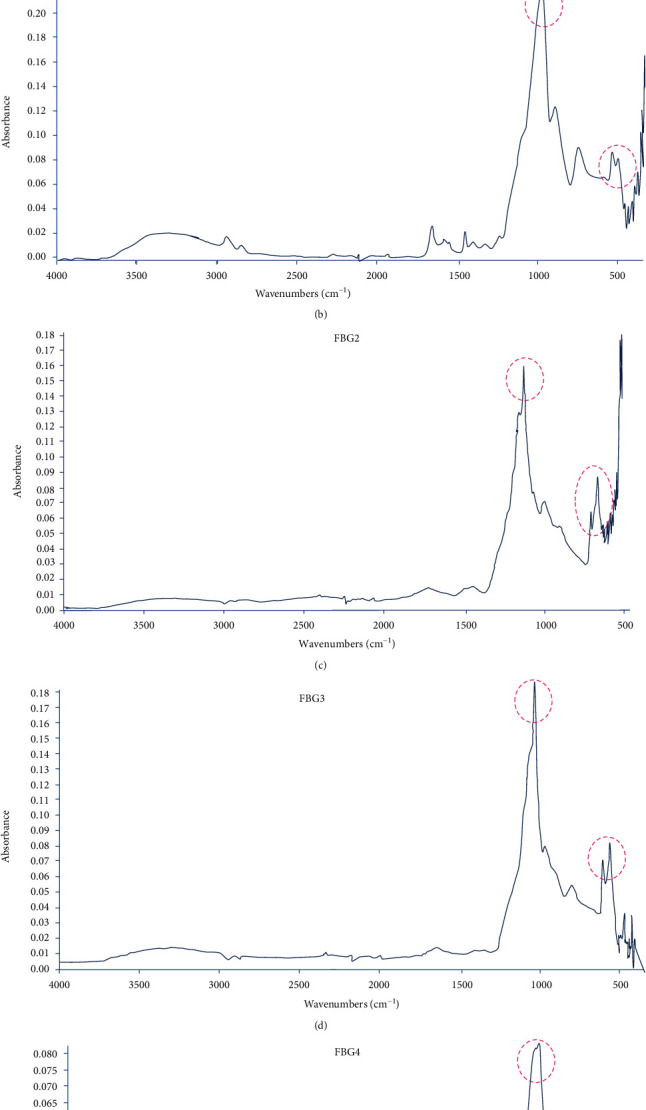
FTIR analysis for the tested groups. The dotted circles are referring to the characteristic hydroxyapatite bands at 560 cm^−1^ and at 1001 cm^−1^. Finger pointer in (e) referring to the single band observed in FBG4.

FTIR spectra obtained from the intact enamel surface before erosion showed the typical FTIR band presence of a sharp (PO_4_^−3^) band at 1040 cm^−1^ and a definite split band at 560 cm^−1^ associated with the hydroxyapatite (Figure 6).

All specimens of the three groups (FBG1-FBG3) treated with variants of fluoride bioactive glasses showed a sharp band at 1040 cm^−1^ and a definite split band observed at 560 cm^−1^. Specimens treated with FBG4 showed single band at 560 cm^−1^ and a sharp band at 1040 cm^−1^ (Figure 6). FTIR examination after erosion showed similar bands to the bands detected before erosion for all groups.

## 4. Discussion

The null hypothesis adopted in the current study was rejected. The “interaction layer” [14–17, 19–21] formed after the FBG application protected the enamel surface (next to orthodontic brackets) through formation of a calcium phosphate layer that resisted a strong erosive challenge.

The experimental design conducted in the current study to test the bioactivity and the interaction of the calcium phosphate-rich layer formed by the different FBG pastes was different from the bioactivity test that was usually conducted through FTIR testing for the sieved particles resulting from FBG discs immersed in Tris buffer solution [25]. Consequently, it may be suggested that the current study simulated more closely the clinical situation. The results showed that mixing the bioactive glass powders with phosphoric acid significantly enhanced the formation of a calcium phosphate-rich layer on top of the treated enamel surface surrounding the orthodontic brackets. The formed calcium phosphate layer resembles the layer that was formed previously by pastes based on 45S5 bioactive glass; however, previous results showed that the layer of calcium phosphate formed was mainly brushite [16] whose crystals needed approximately 14 days of storage in saliva to change to hydroxyapatite [16]. However, the current experiment showed that there is strong evidence for the formation of hydroxyapatite, fluorapatite, and carbonated fluorapatite [25, 26] after 24 hours which was supported by observing the bifid peaks of FTIR spectra at 560 cm^−1^ and the characteristic peak at 1050 cm^−1^ [25, 26]. The second evidence for the suggested formation of apatite phases on top of enamel was confirmed by the chemical characterization of the former layer by FTIR/ATR analysis after the erosion challenge. In the current experiment, a diluted form of citric acid was utilized [23] to test the acid resistance of the interaction layer formed onto the bioactively treated enamel surfaces because citric acid is one of the most widely used components in most of the commercially available soft drinks and juices and is responsible for their erosive potential [23].

Previous research showed that phosphate content in bioactive glasses improved its bioactivity through enhancing the formation of its bioactive calcium phosphate-rich layer [27], which agrees with the obtained results of the current experiment.

This study confirmed the previous results [25] that concluded that sodium component in the fluoride bioactive material is not essential for the bioactivity of the FBG [25] as all pastes were capable of inducing the formation of the bioactive calcium phosphate-rich layer with strong evidence of forming apatite that was confirmed by observing the apatite bands at 560 cm^−1^ and the bands observed at 1001 cm^−1^.

On the other hand, the current research showed that there was a variation in the bioactivity of the tested glasses according to their composition because the FTIR/ATR showed single band at 560 cm^−1^ for the FBG4 (sodium-free FBG) instead of the characteristic split band peaks of the hydroxyapatite that were evident in all sodium-containing FBG.

The single band at 560 cm^−1^ indicated the formation of apatite precursors [25] that may need longer duration to form the apatite stable form [21, 25]. However, acid resistance of the sodium-free FBG glass showed similar results as the rest of the tested pastes.

The suggested mechanism of action for the FBG pastes tested in the current experiment might suggest that the FBGs upon being mixed with phosphoric acid aqueous solution rapidly release high amounts of calcium ions [15]. Also, the silica network of the fluoride bioactive glass would break down and form the water-soluble silanol compounds [17, 28] which would be completely washed from the formed interaction layer upon washing it with strong water spray after 24 hours as was demonstrated previously. The calcium ions released from the fluoride bioactive glass would combine with the phosphate ions released from the phosphoric acid solution to form calcium phosphate compounds that were observed by FTIR/ATR in the current experiment.

Previous studies showed that increasing the phosphate or calcium content of the storage solution in which bioactive glasses are placed will improve the crystallinity and the bioactive reaction of the bioactive glass [26, 29, 30]. Thus, it was expected that storing the specimens in artificial saliva will increase the stability and the crystallization of the formed hydroxyapatite layer. However, orthodontic patients develop cariogenic biofilm rapidly [5, 31] due to abundance of retentive areas around the orthodontic brackets, which makes the oral condition of orthodontic patients and resembles the oral condition of high-caries-risk patients.

In the current experiment, we adopted a protocol [17, 18, 22, 32] for storing the in vitro specimens in which specimens were stored in distilled water for 24 hours which was free from the rich content of calcium and phosphate to examine the bioactivity of the tested bioactive glasses under condition of deprivation from calcium and phosphate ions. Moreover, recent study showed the antibacterial potential of the fluoride bioactive glass suggesting the possible decrease of bacterial content of oral biofilm surrounding the orthodontic brackets [33].

The obtained results of the current experiment confirmed the capacity of the tested bioactive sealers to complete its bioactive cycle independent of the existence of important minerals and thus resembling the condition of high-caries-risk patients and orthodontic patients whose saliva is rich in streptococcus mutans [7] with low buffering capacity and ingesting considerable amounts of erosive drinks.

**Figure 7:**
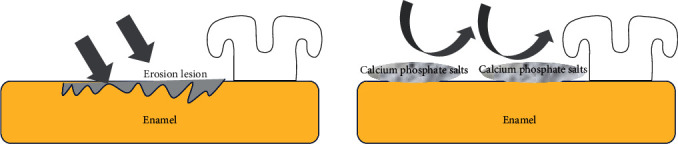
Schematic illustration for the action of the FBG as a suggested orthodontic sealer.

In a previous study [15], the fluoride-containing bioactive glasses remineralized enamel within an extremely short time. Thus, it may be suggested that fluoride bioactive glass pastes used in the current study had strong potentials to exert a dual protective/remineralizing effect on early caries lesions that may develop either in orthodontic patients or in high-caries-risk patients (Figure 7). Further confirmatory study should be conducted using the X-ray diffraction analysis to confirm the crystalline nature of the formed layer.

## 5. Conclusion

Within the limitations of the current in vitro study, it may be concluded that fluoride bioactive glass pastes in this experiment could form a protective interaction layer on enamel surface capable of decreasing the risk of eroding enamel, suggesting its possible use as an enamel sealer capable of protecting enamel in orthodontic and in high-caries-risk patients.

## Figures and Tables

**Table 1:** Compositions of bioactive glasses in mol%, melting (*T*_m_), glass transition (*T*_g_), and crystallization peak temperature (*T*_x_) in °C.

	SiO_2_ (mol%)	CaO (mol%)	P_2_O_5_ (mol%)	Na_2_O (mol%)	CaF_2_ (mol%)	*T*_m_	*T*_g_	*T*_x_
FBG1	37	48.9	6.1	5	3	1500	670	822
FBG2	37	46.4	6.1	7.5	3	1500	640	808
FBG3	37	43.9	6.1	10	3	1450	602	789
FBG4	37	53.9	6.1	0	3	1550	730	864

**Table 2:** Resin materials used in this study.

Material (manufacturer)	Composition
PALFIQUE Bond (Tokuyama Dental, Japan)	Phosphoric acid monomer, bisphenol A di(2-hydroxy propoxy) dimethacrylate (Bis-GMA), triethylene glycol dimethacrylate, 2-hydroxyethyl methacrylate (HEMA), camphorquinone, alcohol, and purified water
Transbond XT light cure adhesive primer (3M Unitek, USA)	Triethylene glycol dimethacrylate, bisphenol A diglycidyl ether, dimethacrylate, triphenylantimony, 4-(dimethylamino)-benzene ethanol, Dl-camphorquinone, and hydroquinone
Transbond PLUS color change adhesive (3M Unitek, USA)	Silane-treated glass, silane-treated quartz, polyethylene glycol dimethacrylate, butanoic acid, 2-hydroxy-4-[[2-[(2-methyl1-oxo-2-propen-1-yl)ox]ethyl]amino]-2-[2[[2-[(2-methyl-1-oxo-2-propen-1yll)oxy]ethyl]amino]-2-oxoethyl]-4-oxo, silane-treated silica, bisphenol A diglycidyl ether, dimethacrylate (Bis-GMA), diphenyliodonium, and hexafluorophosphate

## Data Availability

The data used to support the findings of this study are available from the corresponding author upon request.
